# Scene-level movie data from Amazon X-Ray in the US market combined with IMDb

**DOI:** 10.1038/s41597-026-06602-y

**Published:** 2026-01-20

**Authors:** Safal Shrestha, Yeonie Heo, Alexander T. J. Barron, Minsu Park

**Affiliations:** New York University Abu Dhabi, Abu Dhabi, UAE

**Keywords:** Arts, Sociology, Communication

## Abstract

This paper presents a structured, scene-level dataset of movie content that addresses the limitations of previous research relying on small or non-standardized screenplay collections. Such collections often lack consistent scene representations and actor metadata and use draft versions that differ from their final cinematic products, limiting both the scale and accuracy for content-level analysis. To overcome these limitations, we compile scene breakdowns for 3,265 movies from Amazon X-Ray in the US Amazon Prime Video market, detailing the characters appearing in each scene and linking them to their corresponding IMDb IDs. Subtitles are included for the subset of 3,110 movies, providing complementary dialogue-level data, and each title is linked to its corresponding IMDb ID to enable augmentation with additional metadata for extended analyses. Integration of these resources can allow accurate, large-scale analyses of on-screen representation, character interactions, and narrative structure that were not feasible with earlier screenplay-based datasets. This dataset enhances the consistency and accessibility of movie data, providing a reliable stepping stone for quantitative research on film as cultural artifacts.

## Background & Summary

Movies are one of the most influential forms of cultural expression, playing a critical role in shaping and reflecting societal norms, values, and identities^1,2^. Despite their global reach and cultural significance, research on films has been largely limited to genre-level or metadata-based analysis, lacking the depth of content-level examination that other art forms have enjoyed. Literature, music, and visual arts have benefited from detailed, large-scale analyses, ranging from textual analysis in literature and lyrics^3,4^ to acoustic and visual analysis in music^5,6^ and art^7,8^. These content-driven methodologies have enabled deeper exploration of themes, narratives, and societal impact across time and space^3,5,6,8–11^. However, movies, which are equally or even more widespread and accessible than these other cultural forms, have not received comparable analytical attention. This gap has hindered our ability to fully leverage films as complex social and cultural artifacts, largely due to the limited availability of comprehensive and accurate content-level data sources.

Existing sources for content-level film analysis, such as screenplays^12,13^ and large-scale subtitle corpora^14^, have provided useful but incomplete insights. Screenplays offer rich narrative details, including dialogues, scene descriptions, and technical notes, but often exist only as early drafts that diverge from the final cinematic product. Subtitles capture spoken dialogue along with speaker labels and sound effects, but omit the visual and non-verbal dimensions of scenes. Critically, both sources lack the ability to accurately map narrative content to precise character identities and temporal boundaries of scenes, limiting the potential for reliable, character- and scene-centric investigations. As a result, previous computational work on film, such as character network or demographic representation analysis^15–22^, has often relied on heuristic or error-prone extraction methods from these text-based resources.

More specifically, many recent studies have employed network abstractions of character interaction, using social network analysis to measure differences in demographic representation^15–18^ and applying advances in graph embeddings^19,20^. These studies typically rely on scene co-occurrence networks extracted from scripts and subtitles^16,20– 23^. However, this approach faces compounding challenges. Beyond the fundamental issue of discrepancies between publicly available scripts and their final filmed versions, the network extraction process itself presents significant difficulties. Character disambiguation remains a well-known problem^15^, making it difficult to reliably construct networks and map characters to rich external metadata, such as actor demographics (e.g., gender, race, age) available on platforms like IMDb.

In this context, Amazon X-Ray provides a unique and reliable source of information that can overcome many of these issues and create complementarity. It contains curated, scene-level information about all visible characters in a movie, including non-speaking roles, thereby enabling precise reconstruction of character (co-)occurrence within each scene. These data are synchronized with on-screen content rather than inferred from textual cues, allowing for more accurate representation of narrative dynamics. Each character is also linked to an IMDb identifier, providing access to rich, structured metadata (e.g., gender, race, and occupation). However, X-Ray metadata lacks IMDb identifiers at the movie level and remains embedded within Amazon’s proprietary ecosystem, limiting its direct reuse for research.

To overcome these limitations, our work contributes in three primary ways: (1) large-scale harvesting and processing of X-Ray data, including scene-level character information and associated subtitles, from the U.S. Amazon Prime Video platform; (2) accurate mapping of movies to their corresponding IMDb identifiers using an automated and validated title- and cast-based matching pipeline; and (3) systematic validation of data coverage and accuracy, providing useful assessments on reproducibility and representativeness across decades and genres.

The dataset includes 3,265 movies with scene-level breakdowns of character appearances, linked to IMDb IDs (on both character- and movie-level). A subset of 3,110 movies additionally includes corresponding subtitles. Specifically, the scene breakdowns derived from Amazon X-Ray provide precise start and end timestamps that delineate the temporal boundaries of each scene with character appearances, enabling clear segmentation of the film’s structure. The subtitle data, in turn, contain start and end timestamps for every line of dialogue, making it straightforward to determine the exact scene in which each line was spoken. Researchers can further enrich the dataset by retrieving additional metadata directly from IMDb using the provided identifiers, enabling a range of analyses spanning representation, screen time, language use, and network structure. Note that although we are unable to include screenplay data due to legal restrictions on redistribution, we provide open-source code that allows researchers to independently expand the dataset by collecting or integrating legally permissible materials.

Our dataset has limitations that warrant acknowledgment. It relies on Amazon’s internal, proprietary processes for X-Ray data generation, and Amazon controls which movies are available at any given time (see the Technical Validation section for the representativeness of the data provided in this Descriptor, relative to award-winning movies by decade). Despite these constraints, we believe this dataset offers substantial net improvements over previous methods and sources and can lead to a significant advancement in film analysis that brings it closer to the depth of exploration that literature, music, and art have long enjoyed. Moreover, as video understanding emerges as a key research area in AI and machine learning^24–27^, while relatively small, this dataset provides a high-quality, structured resource to help advance new computational models and analyses. By making this comprehensive content-level dataset publicly available, we offer researchers a valuable tool to explore underrepresented areas of analysis in the broader domain of culture and creative work.

## Methods

**Fig. 1:**
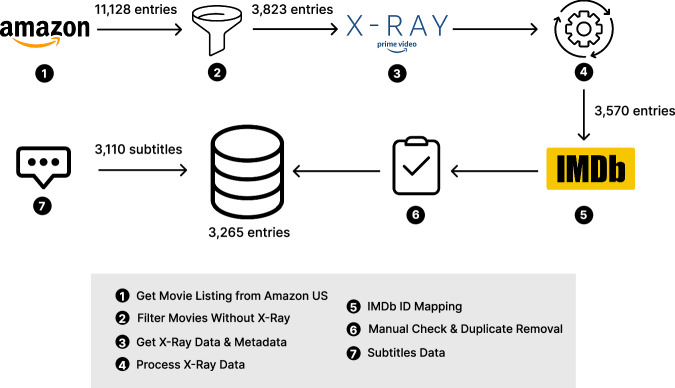
Data retrieval and processing pipeline. This pipeline processes 3,265 X-Ray movies and matches them with 3,110 associated subtitles.

Our data collection pipeline comprised several steps of retrieval and refinement, as illustrated in Fig. 1 and described in this section.

**Fig. 2:**
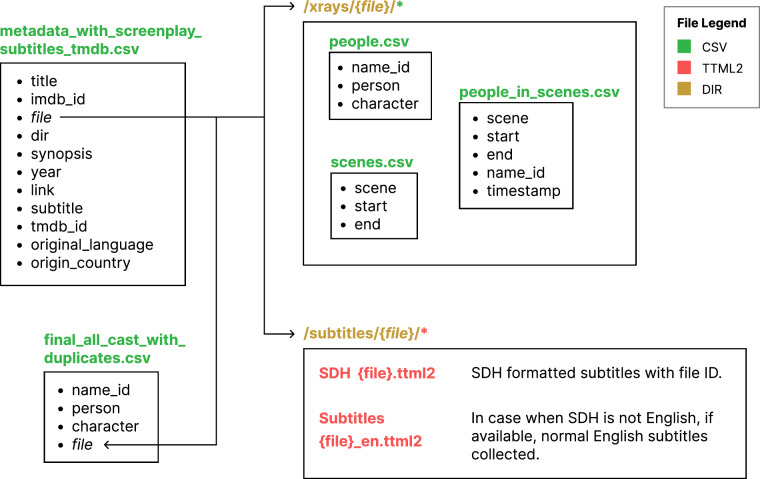
Structure of the augmented Amazon X-Ray Dataset. All X-Ray movie data produced and cleaned through our pipeline are organized by this directory and file schema. Locations of specific data are described in the top-level metadata files metadata_with_subtitles_tmdb.csv, and final_all_cast_with_duplicates.csv, which use keys to index files in the indicated subdirectories. An explanatory notebook data_query_examples.ipynb, included in the dataset repository, shows how to query data with several examples. See Tables 1, 2, and 3 for descriptions of all csv files.

### Retrieval of Movie Entries from Amazon US

#### Defining retrieval scope

Due to intellectual property laws, Amazon Prime Video offers different selections of movies and TV series across various regions. Our data resource focuses on the US market, collecting data from the Amazon US website (https://www.amazon.com/gp/video/storefront) in August 2023. At the time of collection, the US Amazon website featured a catalog of movies and TV series under the Prime Video category. We chose to collect only movies bundled with Prime, which did not incur additional costs beyond the Prime subscription, ensuring the broadest possible audience for the corpus.

#### Retrieving initial data

We used the selenium-wire browser automation library^28^, an extension of selenium^29^ that allows inspection of browser requests and responses, for data collection. The Amazon US website limits pagination, allowing navigation up to page 400. To overcome this limitation, we employed a filtering approach to access all movie entries in successive cohorts. First, we gathered movies marked as “Included with Prime” without applying additional filters. We then expanded this initial collection by filtering movies by their release year in decade-based batches: before 2010, between 2010-2020, and after 2020. Although Amazon’s filtering is not always accurate, this approach increased data recall when merging results across cohorts.

#### Processing entries and duplicates removal

**Fig. 3:**
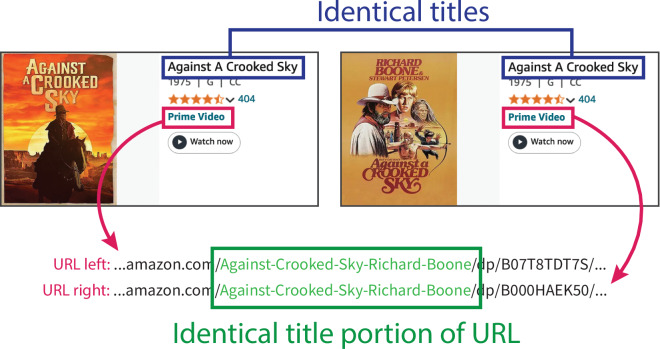
Example of a duplicate movie based on Prime Video URL and title. Since these two entries have identical titles and identical title portions of their URLs, they are considered the same movie.

Each entry we retrieved included its page URL and film title. Through manual inspection, we found that multiple films could share the same title, and a single film could have multiple titles. To remove duplicates, we used the film title and a portion of the unique URL from the Prime Video page, as shown in Fig. 3. Entries were identified as duplicates if they shared the same “title and URL portion” pair. This heuristic successfully de-duplicated most of the movies in our dataset, resulting in 11,128 entries with links to their respective Prime Video pages.

For each entry, we created a unique identifier using the title collected at this step. This identifier, used as a directory name, was constructed by preprocessing the title to remove non-alphanumeric characters with the unidecode library^30^, mapping any non-ASCII characters to ASCII format and replacing spaces with underscores. Each movie identifier was also prefixed with its index within the batch. For example, a movie titled “12 Days with God” was mapped to “1265_12_Days_with_God,” where *1265* is the index and *12_Days_with_God* is the processed title.

### Collection of X-Ray Data with IMDb ID Mapping

#### Filtering movies without X-Ray

**Fig. 4:**
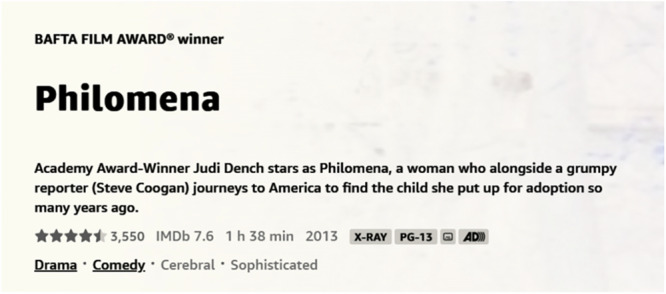
Example of the relevant portion of the Amazon Prime Video page for the movie *Philomena*.

Not all collected listings on Amazon Prime Video contain X-Ray data. We used browser automation tools to visit each movie’s Prime Video page and collect additional metadata on X-Ray availability. As shown in Fig. 4, the Prime Video page includes movie details such as title, description, and tags. The presence of an “X-Ray” tag indicates whether X-Ray data is available. After processing these pages with BeautifulSoup^31^, we excluded movies without the X-Ray tag, reducing the dataset to 3,823 entries.

#### Retrieving X-Ray data and metadata

We collected details for each entry in our list by intercepting two network requests triggered when clicking the play button on each movie. First, we intercepted the request for “PlaybackResource,” whose JSON response contained metadata, including the title, entity type (movies), runtime, synopsis, ratings, subtitle types (subtitle, narrative, or SDH), descriptions, image links, and links to subtitles (in multiple languages, where available). It also included additional information such as audio tracks and additional metadata uniquely available on the Prime Video platform. Second, we intercepted the request for “X-Ray,” whose JSON response contained timestamp information for characters appearing in different scenes. This approach builds on previous work^32^. We saved the responses into two JSON files: *PlaybackResources.json* and *Xray.json*.

#### Processing X-Ray data

We extracted metadata from each movie’s *PlaybackResource* file and compiled it into a single file. We then parsed the X-Ray files into three structured files for each movie, as detailed in Table 1. These X-Ray-derived files include scene boundary timestamps (in *scenes.csv*) and scene-level character appearance information (in *people_in_scenes.csv*), providing precise information on which characters appear on screen. Not all X-Ray files included scene-wise cast appearance data, so we removed entries with missing or incomplete scene-level cast data, leaving 3,570 entries.

**Table 1:** Parsed X-Ray data files.

Field for Data File	Description
**people.csv**: Contains a list of actors, corresponding characters, and IMDb name IDs for actors.	
name_id	Name ID of person from IMDb. The URL corresponding to a name_id would be https://www.imdb.com/name/ < name_id >. Example: https://www.imdb.com/name/nm0451321for *nm0451321*.
person	Name of the person.
character	Name of the character in the movie.
**scenes.csv**: Contains a list of scenes, along with the start and end timestamps of each scene.	
scene	Scene number.
start	Scene start timestamp in milliseconds.
end	Scene end timestamp in milliseconds.
**people_in_scenes.csv**: Contains a list of scenes with IMDb IDs of people appearing in the scene, along with start and end timestamps.	
scene	Scene number.
start	Scene start timestamp in milliseconds.
end	Scene end timestamp in milliseconds.
name_id	Name ID of person from IMDb.
timestamp	Timestamp of the character’s first appearance in the scene, in milliseconds.

These files are provided for each film in the schema shown in Fig. 2 under the xrays directory. Note that the scene timestamps in these files are not known to be aligned to the subtitle timestamps contained in *.ttml2* files (Fig. 2). However, the example Jupyter notebook demonstrates how subtitles can be assigned to scenes based on temporal overlap. The majority of subtitle segments fall fully within scene boundaries, indicating a strong degree of alignment between the two timestamp sources. Perfect correspondence is not expected, as spoken dialogue may cross visual scene boundaries.

Note that we compiled subtitle files (*.ttml2*) for all movies in our metadata list that included them (see Fig. 2). These subtitle files provide start and end timestamps for contiguous subtitles text shown on-screen, allowing researchers to identify the precise time periods when dialogue occurs. When the timing is sufficiently close to or falls within the scene boundaries defined by X-Ray data, these dialogue segments can be associated with the corresponding scene context (see the Jupyter notebook included in the dataset for examples of this).

#### Mapping IMDb IDs

Linking each retrieved X-Ray movie to its corresponding IMDb ID provides a way to enrich our data with background cast, alternative titles, user ratings, crew and cast information, awards, nominations, quotes, and more. Unfortunately, the *PlaybackResource* information does not include IMDb IDs, so we devised an algorithm inspired by Ramakrishna *et al*.^12^ to match movies to their IMDb entries. Since X-Ray data already provides accurate information about the actors, along with their IMDb profiles, we used this data to assist in matching.

Accordingly, we used the *cinemagoer* Python package^33^ to retrieve data from IMDb. We initiated the search using the movie title from its *PlaybackResource* file, which returned several matches from IMDb. We then examined the top 5 cast members from the top 5 movie matches. Previous studies have shown that IMDb cast order reflects the importance of cast members^15,16^, and matching key cast members can clearly distinguish between movies. Additionally, since the X-Ray data is sourced directly from IMDb^34^, matching the top 5 cast proved effective. If at least one actor from this top-5 list matched an actor from the X-Ray data, we recorded the IMDb ID as a match. This process resulted in 3,129 successful matches out of 3,570 movies, leaving 441 unmatched.

We manually mapped the 441 unmatched movies to their correct IMDb IDs, and the procedure is detailed in the Technical Validation section. To verify the overall accuracy of our initial, automated IMDb ID matching process, we randomly sampled 120 movies from the 3,129 matches and found only one matching error, resulting in an error rate of 0.83%. The code for this automated matching process is available on Github at https://github.com/safal312/xray-collector. The final dataset, including all the manually matched entries, comprises 3,265 movies and represents a thoroughly validated and cleaned set of matches. All identified sources of error were corrected, resulting in a dataset that is ready for use^35^ (Fig. 2).

## Data Records

The dataset is available at Zenodo with 10.5281/zenodo.17659734^35^. The processed and cross-referenced X-Ray data files described in the Methods section are outlined in Fig. 2. The parsed X-Ray data, metadata, and cast data files are provided in *.csv* or *.txt* format and are detailed in Tables 1, 2, and 3. A Jupyter notebook containing examples of how to query the dataset is included in the data repository.

**Table 2:** Fields in X-Ray movie metadata file metadata_with_subtitles_tmdb.csv, one of the top-level files in the schema shown in Fig. 2.

Field	Description
title	Title of the movie.
imdb_id	IMDb ID of the movie.
file	Unique identifier of the movie in the dataset.
dir	Name of the batch in which the data was collected (e.g., *com*, *before2010*, *in2010s*, *after2020*). Data was collected in batches using decade-based filtering. The “com” batch is the initial collection without filters.
synopsis	Brief synopsis of the movie, collected from Prime Video.
year	Year of the movie’s release.
link	Link to the Prime Video page of the movie; all must have the prefix www.amazon.com added to form the full URL.
subtitle	Indicator of subtitle availability:
SDH	Movie has English SDH subtitle.
SDH_EN	Movie has non-English SDH and English non-SDH subtitles.
EN	Movie only has English non-SDH subtitle.
Null	Subtitle data not available.
tmdb_id	TMDb ID of the movie (identifier for https://www.themoviedb.org/, an alternative movie database).
original_language	Language of origin of the movie, sourced by TMDb. Null if data unavailable.
original_country	Country of origin of the movie. Null if data unavailable.

**Table 3:** Fields in cast metadata file final_all_cast_with_duplicates.csv, one of the top-level files in the schema shown in Fig. 2.

Field	Description
name_id	IMDb ID of the person.
person	Name of the person.
character	Character name in the movie.
file	Unique indicator of the movie in the dataset.

“Duplicates” refer to the fact that one person can have multiple castings across different movies, and therefore multiple rows in this file.

## Technical Validation

### Validation and Resolution of Errors

We conducted technical validation for two sets of movies: (1) those successfully matched with their corresponding IMDb IDs and (2) those that initially remained unmatched.

For the first set of 3,129 movies successfully matched to IMDb IDs, we assessed the accuracy of the matching process as described in the “Mapping IMDb IDs” section. We randomly sampled 120 movies from this set and found only one matching error, resulting in an error rate of 0.83%, which suggests high accuracy and reliability for our automated matching process.

For the 441 unmatched movies, we manually mapped each movie to the correct IMDb ID, aiming to maximize accuracy. In this process, we also identified the reasons for the initial matching failures. First, using the movie title and additional metadata (e.g., description, release year, and cast), we retrieved IMDb IDs for each entry, regardless of whether they were classified strictly as movies. During this manual verification, we identified several non-movie entries (e.g., stand-up comedy specials and anthologies) mistakenly included due to Amazon Prime Video’s classification errors. These entries (*N* = 20), which do not follow traditional narrative movie structures, were excluded from the final dataset.

Further investigation of the 441 unmatched entries revealed that some PlaybackResource and X-Ray files did not correspond to the listed movie. In a subset of cases (28 PlaybackResource files and 94 X-Ray files), the Amazon US movie pages returned erroneous files that were duplicates of other movie listings. To identify these cases, we compared the unique movie ID generated at the start of our data pipeline with the ID generated from the retrieved PlaybackResource and X-Ray files. For the movies with erroneous PlaybackResource files, IMDb matching was impossible because the title used in the search did not align with the actual cast list, so we manually corrected the metadata for these entries. For the erroneous X-Ray files, IMDb matching was not feasible due to the lack of reliable cast data, so we removed these entries entirely.

Finally, we applied these insights from the unmatched cohort to the larger, automatically matched set to identify any remaining discrepancies in X-Ray and PlaybackResource files. After either correcting or removing problematic entries, we compiled a clean and complete final dataset of 3,265 movies.

### Coverage Assessment

The applicability of this dataset depends on its coverage and representativeness of the full range of films produced throughout history. We evaluate this coverage in two ways: by comparing our dataset against IMDb lists of the 100 most popular movies per decade and against lists of Academy Award-winning films by year, sourced from the Academy Awards official website (Table 4). Coverage is sparse in the earlier decades, with only select years, such as 1931, 1932, 1936, and 1939, represented in the 1930s. However, from the 1950s onward, our dataset includes movies from each year, showing progressive improvement in coverage over the decades.

**Table 4:** Decadal coverage of the *Augmented Amazon X-Ray Dataset* for the top-100 most popular movies and Academy Awards.

Decade	Years covered in dataset	Total movies in dataset	Screenplays in top 100 list by decade	Academy Award-winning movies (total awards given)
1930s	1931, 1932, 1936, 1939	6	1	0 (106)
1940s	1940, 1941, 1944, 1945, 1947, 1948, 1949	10	2	1 (180)
1950s	All years	24	7	4 (154)
1960s	All years	34	5	5 (147)
1970s	All years	54	11	6 (134)
1980s	All years	91	3	1 (136)
1990s	All years	145	4	7 (139)
2000s	All years	327	10	8 (147)
2010s	All years	1,403	9	19 (149)
2020s	All years	1,171	7	3 (56)
**Total**	—	3,265	59 (of 939)	54 (of 1,348)

There are a total of 939 possible top-100 movies per decade, instead of 1,000, because IMDb provided only 39 for the 2020s.

Although our datasets’ coverage is moderate relative to these benchmarks, this presents a valuable analytical opportunity: the ability to study films that may not be well-remembered or acclaimed as the best of their time. By sampling movies based on production rather than popularity, our dataset mitigates survival bias, providing a more representative selection of films. This broad coverage, combined with the dataset’s unique scene-level breakdown, is a resource not previously available in film studies.

To further enhance coverage, especially for recent decades, we plan to implement periodic updates. These updates will involve collecting additional data as it becomes available and refining our collection methods to capture more recent releases. The code for this process is available for others to use as well. Additionally, exploring partnerships with movie databases and production companies could provide better access to recent, high-quality metadata. This proactive approach will help ensure that our dataset remains a dynamic and valuable resource for cultural analysis and film studies.

## Data Availability

The dataset is available at Zenodo with 10.5281/zenodo.17659734^35^.

## Code availability

Code is available on Github at https://github.com/safal312/xray-collector.

## References

[1] Belton, J.*Movies and mass culture* (Bloomsbury Publishing, 1996).

[2] Grindstaff, L. & Turow, J. Video cultures: Television sociology in the “new tv” age. *Annual Review of Sociology* **32**, 103–125 (2006).

[3] Reagan, A. J., Mitchell, L., Kiley, D., Danforth, C. M. & Dodds, P. S. The emotional arcs of stories are dominated by six basic shapes. *EPJ data science* **5**, 1–12 (2016).

[4] Park, M., Park, J., Rojas, F. & Ahn, Y.-Y. Rap music as a social reflection: Exploring the relationship between social conditions and expressions of violence and materialism in rap lyrics. *SocArXiv* (2024).

[5] Park, M., Thom, J., Mennicken, S., Cramer, H. & Macy, M. Global music streaming data reveal diurnal and seasonal patterns of affective preference. *Nature Human Behaviour* **3**, 230–236 (2019). 30953008 10.1038/s41562-018-0508-z

[6] Lee, H. *et al*. Global music discoveries reveal cultural shifts during the war in ukraine. *PsyArXiv* (2024).

[7] Liu, L., Dehmamy, N., Chown, J., Giles, C. L. & Wang, D. Understanding the onset of hot streaks across artistic, cultural, and scientific careers. *Nature Communications* **12**, 5392 (2021). 34518529 10.1038/s41467-021-25477-8 PMC8438033

[8] Lee, K., Park, J., Goree, S., Crandall, D. & Ahn, Y.-Y. Social signals predict contemporary art prices better than visual features, particularly in emerging markets. *Scientific Reports* **14**, 11615 (2024). 38773156 10.1038/s41598-024-60957-z PMC11109285

[9] McDonnell, T. E. Cultural objects, material culture, and materiality. *Annual Review of Sociology* **49**, 195–220 (2023).

[10] Park, M., Weber, I., Naaman, M. & Vieweg, S. Understanding musical diversity via online social media. In *Proceedings of the International AAAI Conference on Web and Social Media*, vol. 9, 308–317 (2015).

[11] Park, M., Park, J., Baek, Y. M. & Macy, M. Cultural values and cross-cultural video consumption on youtube. *PLoS ONE* **12**, e0177865 (2017). 28531228 10.1371/journal.pone.0177865 PMC5439684

[12] Ramakrishna, A., Martínez, V. R., Malandrakis, N., Singla, K. & Narayanan, S. Linguistic analysis of differences in portrayal of movie characters. In *Proceedings of the 55th Annual Meeting of the Association for Computational Linguistics (Volume 1: Long Papers)*, 1669–1678 (2017).

[13] Gorinski, P. J. & Lapata, M. Movie Script Summarization as Graph-based Scene Extraction. *Proceedings of the 2015 Conference of the North American Chapter of the Association for Computational Linguistics: Human Language Technologies*. p. 1066–1076, (Eds. Rada Mihalcea, Joyce Chai, Anoop Sarkar) 10.3115/v1/N15-1113 (Gorinski & Lapata, NAACL 2015).

[14] Davies, M. *The Corpus of Contemporary American English (COCA)*. Available online at https://www.english-corpora.org/coca/ (2008).

[15] Kagan, D., Chesney, T. & Fire, M. Using data science to understand the film industry’s gender gap. *Palgrave Communications* **6**, 1–16 (2020).

[16] Tran, Q. D. & Jung, J. E. Cocharnet: Extracting social networks using character co-occurrence in movies. *J. Univers. Comput. Sci.* **21**, 796–815 (2015).

[17] Malik, M., Hopp, F. R. & Weber, R. Representations of Racial Minorities in Popular Movies. *Computational Communication Research* **4**, 10.5117/CCR2022.1.006.MALI (2022).

[18] Agarwal, A., Zheng, J., Kamath, S., Balasubramanian, S. & Dey, S. A. Key female characters in film have more to talk about besides men: Automating the bechdel test. In *Proceedings of the 2015 Conference of the North American Chapter of the Association for Computational Linguistics: Human Language Technologies*, 830–840 (2015).

[19] Lee, O.-J. & Jung, J. J. Story embedding: Learning distributed representations of stories based on character networks. *Artificial Intelligence* **281**, 103235, 10.1016/j.artint.2020.103235 (2020).

[20] Mourchid, Y. *et al*. Movienet: a movie multilayer network model using visual and textual semantic cues. *Applied Network Science* **4**, 121, 10.1007/s41109-019-0226-0 (2019).

[21] Kaminski, J., Schober, M., Albaladejo, R., Zastupailo, O. & Hidalgo, C. Moviegalaxies - Social Networks in Movies, 10.7910/DVN/T4HBA3 (2018).

[22] Agarwal, A., Balasubramanian, S., Zheng, J. & Dash, S. Parsing screenplays for extracting social networks from movies. In *Proceedings of the 3rd Workshop on Computational Linguistics for Literature (CLFL)*, 50–58 (2014).

[23] Lee, O.-J., Jo, N. & Jung, J. J. Measuring character-based story similarity by analyzing movie scripts. In *Text2Story@ ECIR*, 41–45 (2018).

[24] Ju, X. *et al*. Miradata: A large-scale video dataset with long durations and structured captions. *Advances in Neural Information Processing Systems* **37**, 48955–48970 (2024).

[25] Zhang, Q., Yue, Z., Hu, A., Wang, Z. & Jin, Q. MovieUN: A dataset for movie understanding and narrating. In Goldberg, Y., Kozareva, Z. & Zhang, Y. (eds.) *Findings of the Association for Computational Linguistics: EMNLP 2022*, 1873–1885, 10.18653/v1/2022.findings-emnlp.135 (Association for Computational Linguistics, Abu Dhabi, United Arab Emirates, 2022).

[26] Chen, L. *et al*. Sharegpt4video: Improving video understanding and generation with better captions. *Advances in Neural Information Processing Systems* **37**, 19472–19495 (2024).

[27] Kayal, P., Mettes, P., Dehmamy, N. & Park, M. Large language models are natural video popularity predictors. In Che, W., Nabende, J., Shutova, E. & Pilehvar, M. T. (eds.) *Findings of the Association for Computational Linguistics: ACL 2025*, 11432–11464, 10.18653/v1/2025.findings-acl.597 (Association for Computational Linguistics, Vienna, Austria, 2025).

[28] Selenium wire. https://pypi.org/project/selenium-wire/. Accessed: August 2023.

[29] Selenium. https://www.selenium.dev/. Accessed: August 2023.

[30] Unidecode. https://pypi.org/project/Unidecode/. Accessed: August 2023.

[31] Beautifulsoup. https://beautiful-soup-4.readthedocs.io/en/latest/. Accessed: August 2023.

[32] Poggel, L. & Fischer, F. Automatic extraction of network data from amazon prime videos (using ‘1917’ as an example). https://weltliteratur.net/extracting-network-data-from-amazon-prime-videos/ (2022).

[33] Cinemagoer. https://cinemagoer.github.io/. Accessed: September 2023.

[34] Introducing ‘x-ray for movies,’ powered by imdb and available exclusively on the all-new kindle fire family.*Amazon.com press center* (2012).

[35] Shrestha, S., Heo, Y., Barron, A. T. & Park, M. Scene-level movie data from Amazon X-Ray in the us market combined with IMDb, 10.5281/zenodo.17659734 (2025). 10.1038/s41597-026-06602-y PMC12920774 41559118

